# Inhibition by Anandamide of 6-Hydroxydopamine-Induced Cell Death in PC12 Cells

**DOI:** 10.1155/2010/818497

**Published:** 2010-02-16

**Authors:** Katarzyna Mnich, David P. Finn, Eilis Dowd, Adrienne M. Gorman

**Affiliations:** ^1^School of Natural Sciences, National University of Ireland, Galway, Ireland; ^2^School of Medicine, National University of Ireland, Galway, Ireland

## Abstract

6-hydroxydopamine (6-OHDA) is a selective neurotoxin that is widely used to investigate cell death and protective strategies in models of Parkinson's disease. Here, we investigated the effects of the endogenous cannabinoid, anandamide, on 6-OHDA-induced toxicity in rat adrenal phaeochromocytoma PC12 cells. Morphological analysis and caspase-3 activity assay revealed that anandamide inhibited 6-OHDA-induced apoptosis. The protection was not affected by antagonists of either cannabinoid receptors (CB_1_ or CB_2_) or the vanilloid receptor TRPV1. Anandamide-dependent protection was reduced by pretreatment with LY294002 (inhibitor of phosphatidylinositol 3-kinase, PI3K) and unaffected by U0126 (inhibitor of extracellularly-regulated kinase). Interestingly, phosphorylation of c-Jun-NH2-terminal kinase (JNK) in cells exposed to 6-OHDA was strongly reduced by anandamide pre-treatment. Furthermore, 6-OHDA induced c-Jun activation and increased Bim expression, both of which were inhibited by anandamide. Together, these data demonstrate antiapoptotic effects of anandamide and also suggest a role for activation of PI3K and inhibition of JNK signalling in anandamide-mediated protection against 6-OHDA.

## 1. Introduction

In recent years, the endogenous cannabinoid (endocannabinoid) system has emerged as a potential therapeutic target for the treatment of Parkinson's disease [1–5]. These studies suggest that the potential therapeutic benefits of cannabinoid drugs may include neuroprotection of nigrostriatal dopaminergic neurons. This is of particular interest since neuroprotective therapies for Parkinson's disease are notably lacking, and current therapies are generally dopamine-enhancing strategies, that neither halt nor delay ongoing neurodegeneration. Anandamide (also known as arachidonylethanolamide), was the first endocannabinoid to be discovered, is derived from arachidonic acid and found principally in brain tissue [6]. Anandamide binds and activates the cannabinoid receptors (CB_1_ and CB_2_) and also the vanilloid receptor, TRPV1 [7, 8].

Mounting evidence supports a role for anandamide in the modulation of cell fate, including cell death and survival. Anandamide can protect neurons from toxic insults such as glutamatergic excitotoxicity, nutrient deprivation, hypoxia and ischemia [9–12]. These protective effects of anandamide have been reported to be mediated by CB_1_ and CB_2_ cannabinoid receptors, whereas activation of TRPV1 has been suggested to mediate anandamide-induced apoptosis in rat C6 glioma cells, human DAUDI leukemia cells, and cervical carcinoma cell lines [13–15]. 

The present study was undertaken to examine the ability of anandamide to protect PC12 cells against 6-hydroxydopamine (6-OHDA) toxicity. 6-OHDA is a hydroxylated analogue of dopamine that is commonly used in model systems to mimic Parkinson's disease [16, 17]. 6-OHDA induces apoptosis of primary mesencephalic dopaminergic neurons [18, 19], MN9D [20] and dopaminergic cell lines including PC12 [17, 21, 22]. Apoptosis is a highly regulated form of cell death that occurs under physiological and pathological conditions. It is characterised morphologically by cell shrinkage and nuclear condensation. These changes are mediated by activation of caspase proteases, and in the case of 6-OHDA this occurs as a result of release of cytochrome *c* from the mitochondria [22]. 

Here we examine the effect of anandamide on 6-OHDA-induced toxicity in PC12 cells. In particular, the mechanism of anandamide action against 6-OHDA was tested by examining the possible role of signalling pathways, which are well known to be involved in regulation of cell fate, including phoshpatidylinositol 3-kinase (PI3K)/Akt, mitogen activated protein kinase (MAPK)/extracellular signal-regulated kinase1/2 (ERK1/2) and c-Jun-NH2-terminal kinase (JNK)/c-Jun pathways.

## 2. Experimental Procedures

### 2.1. Materials

Rat phaeochromocytoma PC12 cells were obtained from European Collection of Cell Cultures (ECACC). All chemicals were supplied by Sigma–Aldrich unless stated otherwise. Anandamide and SB366791 were obtained from Tocris Bioscience. SR141716A and SR144528 were from NIMH Chemical Synthesis and Drug Supply Program. U0126 and SP600125 were supplied by Calbiochem. Rabbit polyclonal antibody against Bim was from StressGen Biotechnologies. Mouse monoclonal antibody against p-JNK, rabbit polyclonal anti-caspase-3 antibody and rabbit monoclonal antibody against p-ERK1/2 were obtained from Cell Signalling Technology. Mouse monoclonal antibody against p-c-Jun was from Santa Cruz Biotechnology. Anti-Actin rabbit polyclonal antibody was from Sigma-Aldrich. Goat secondary antibodies conjugated to horseradish peroxidase were from Pierce. Ac-Asp-Glu-Val-Asp-a-(4-methyl-coumaryl-7-amide) (DEVD-MCA) was from the Peptide Institute, Osaka, Japan. Protein molecular weight marker was obtained from New England Biolabs.

### 2.2. Cell Culture and Treatments

Rat adrenal phaeochromocytoma PC12 cells were cultured in DMEM supplemented with 10% horse serum, 5% fetal calf serum, 50 U/ml penicillin and 50 *μ*g/ml streptomycin, at 37°C in a humidified 5%  CO_2_ atmosphere. For experiments, cells were seeded at a density of 7 × 10^4^ cells/cm^2^ in plates coated with 10 *μ*g/ml poly-L-lysine. Cells were left overnight before commencing experimental treatments. Stock solutions of 6-OHDA were made freshly in sodium metabisulfite (1 M) prior to each experiment. Unless otherwise stated, PC12 cells were incubated with 25 *μ*M anandamide for 24 hours followed by treatment with 100 *μ*M 6-OHDA for further 24 hours before analysis.

### 2.3. Cell Viability and Proliferation Assay

Cell survival was determined by using colorimetric MTT (3-(4,5-dimethylthiazolyl-2)-2,5-diphenyltetrazoliumbromide) tetrazolium salt assay [23]. PC12 cells were plated into 96-well plates at 1.7 × 10^4^ cells/well in 100 *μ*l of medium. MTT tetrazolium salt was dissolved in Hank's balanced salt solution to concentration of 5 mg/ml. After experimental treatments, 10 *μ*l of MTT was added to the culture for 3 hours at 37°C. To stop the reaction and solubilize the formazan crystals 100 *μ*l of 20% SDS in 50% dimethyl formamide was added and the absorbance was measured at 550 nm by a Wallac 1420 plate-reader with a reference wavelength of 650 nm. Cell viability was expressed as percent of the control culture.

### 2.4. DAPI Staining of Nuclei

Cells were washed in PBS and fixed with 3.7% paraformaldehyde for 10 minutes at room temperature. Nuclei were stained with 4′-6-diamidino-2-phenylindole (DAPI) and placed in mounting medium (Vectashield, Vector, Burlingame, CA). 5 *μ*l aliquot of the cell suspensions were applied to glass slides. Morphological changes in chromatin of cells undergoing apoptosis were analysed by fluorescence microscopy (excitation 350 nm and emission at 460 nm). Cells were scored by counting at least 300 cells from each sample.

### 2.5. DEVDase Activity Assay

The activity of caspase-3-like enzymes (DEVDases) was determined fluorometrically as reported previously [24] with some modifications [22, 25]. Briefly, cells were scraped and spun down at 300 × g at 4°C for 5 min. Pellets were washed in ice-cold phosphate-buffered saline (PBS) and spun down again at 20 000 × g for 10 seconds. The pellets were re-suspended in 25 *μ*l PBS and snap-frozen in liquid nitrogen. 50 *μ*M of DEVDase-substrate (DEVD-MCA) in reaction buffer (100 mM N-2-hydroxyethyl-piperazine-N-2-ethanesulphonic acid (HEPES) pH 7.5, 10% sucrose, 0.1% 3-[(3-cholamidopropyl)-dimethylammonio]-1-propanesulfonate (CHAPS), 5 mM dithiothreitol (DTT), 10^−4^% Nonidet-P-40) was added into lysates and the release of free AMC was monitored at 37°C at 60 seconds intervals over a 30 minute period using a Wallac Victor multilabel counter (excitation 355 nm, emission 460 nm). Fluorescent units were converted to nanomoles of AMC released using a standard curve generated with free AMC and subsequently related to protein concentration.

### 2.6. Preparation of Whole Cell Extracts

Following experimental treatments cells were scraped from the culture flasks and centrifuged at 150 × g for 5 minutes at 4°C. After washing in PBS cells were lysed using whole cell lysis buffer (20 mM HEPES pH 7.5, 350 mM NaCl, 1 mM MgCl_2_, 0.5 mM EDTA, 0.1 mM EGTA, 1% Nonidet P-40, 0.5 mM DTT, 0.1% phenylmethylsulphonyl fluoride (PMSF), 1% aprotinin, 5 mM NaF, and 1 mM Na_3_VO_4_). Cellular debris was spun down at 20,000 × g for 1 minute and supernatant was taken to determine the protein content using Bradford reagent with bovine serum albumin (BSA) as the standard.

### 2.7. Western Blotting

40 *μ*g of proteins were denatured using Laemmli's sample buffer (62 mM Tris-HCl, pH 6.8, 2% sodium dodecyl sulphate (SDS), 5%  *β*-mercaptoethanol, 4% glycerol, 1 mM PMSF, 0.01% bromophenol blue) and boiled at 95°C for 5 minutes. Proteins were separated by 10%–15% SDS-PAGE and electrophoretically transferred onto nitrocellulose membranes. Membranes were blocked for 1 hour in PBS containing 0.05% Tween 20 and 5% (w/v) non-fat dried milk. Membranes were probed with antibodies (1  :  1000) overnight at 4°C followed by appropriate horseradish peroxidise-conjugated goat secondary antibody at 1  :  10,000 (or 1  :  2,000 for detection of caspase-3) for 2 hours at room temperature. Protein bands were visualized using Supersignal West pico system (Pierce).

### 2.8. Statistical Analysis

Values are expressed as means ± SEM of 3 separate experiments unless otherwise indicated. Statistical analysis was performed using repeated-measures ANOVA followed by Bonferroni multiple comparisons *post hoc* test, for which levels of *P* < .05 were considered to be significant.

## 3. Results

### 3.1. Anandamide Pre-Treatment Protects PC12 Cells against 6-OHDA Toxicity

**Figure 1:**
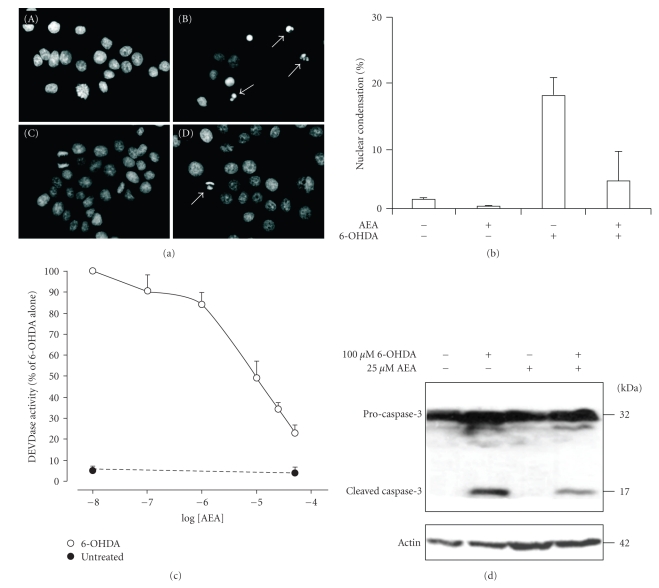
Inhibition of 6-OHDA-induced apoptosis by anandamide. (a) PC12 cells were treated with 25 *μ*M anandamide for 24 hours and then exposed to 100 *μ*M 6-OHDA for a further 24 hours. Cytocentrifuge preparations of control and treated cells were stained with DAPI to visualise nuclear changes. The apoptotic cells with condensed and fragmented nuclei are indicated by arrows. Cells were (a) untreated, (b) exposed to 6-OHDA, (c) treated with AEA only or (d) treated with AEA and then exposed to 6-OHDA. (b) The level of cell death due to 6-OHDA with and without prior anandamide treatment was quantified and expressed as a percentage of the total number of cells. The results shown are the average of two separate experiments ± range. (c) PC12 cells were treated with a range of concentrations of anandamide (0–50 *μ*M) for 24 hours prior to exposure to 100 *μ*M 6-OHDA for a further 24 hours. DEVDase activity was measured in whole cell extracts. Values represent the mean ± SEM of four independent determinations. (d) PC12 cells were exposed to 25 *μ*M anandamide for 24 hours followed by treatment with 100 *μ*M 6-OHDA for further 24 hours. Pro-caspase-3 processing was visualized by Western blotting. Actin was used as a loading control. The data are representative of two independent experiments.

We have previously shown that 6-OHDA causes a concentration-dependent induction of apoptosis in PC12 cells [22]. In order to examine the effect of anandamide against 6-OHDA, PC12 cells were incubated with 25 *μ*M anandamide for 24 hour followed by exposure to 100 *μ*M 6-OHDA for a further 24 hours. Visualisation of nuclear morphology by DAPI staining demonstrated that pre-treatment of PC12 cells with anandamide inhibited 6-OHDA-induced apoptosis (Figure 1(a)). The level of cell death due to 6-OHDA with and without prior anandamide treatment was quantified and expressed as a percentage of the total number of cells. Anandamide alleviated the morphological manifestation of cell damage and reduced cell death from 19.2 ± 2.8% to  4.7 ± 4.6% (Figure 1(b)).

Next the effect of anandamide on caspase 3-like activity (DEVD-MCA cleavage activity) was examined. PC12 cells were treated with 0–50 *μ*M anandamide for 24 hours prior to treatment with 6-OHDA for a further 24 hours. Anandamide effectively inhibited DEVD-MCA-cleavage activity in a concentration-dependent manner, causing approximately 50% inhibition at 10 *μ*M anandamide (Figure 1(c)). This was accompanied by a reduction in the processing of pro-caspase-3 into its active 17 kDa form, as detected by Western blotting (Figure 1(d)). These data indicate that anandamide can protect against 6-OHDA-induced apoptosis at the level of, or upstream of, caspase-3 activation.

### 3.2. Anandamide-Mediated Protection Is Cannabinoid and Vanilloid Receptor-Independent

**Figure 2:**
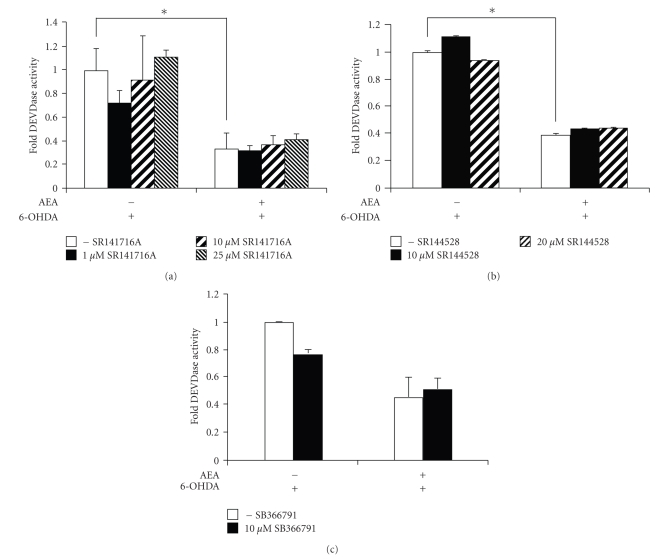
Anandamide-mediated prosurvival effect is cannabinoid and vanilloid receptor-independent. PC12 cells were treated with 25 *μ*M anandamide for 24 hours, with or without selective receptor antagonists, and then exposed to 100 *μ*M 6-OHDA for a further 24 hours. Effector caspase activity was measured by DEVDase assay in whole cell extracts. (a) Indicated concentrations of CB_1_ receptor antagonist SR141716A were added 1 hours prior to incubation with anandamide. Values represent the mean ± SEM of 3 independent determinations, *P < .001. (b) Indicated concentrations of CB_2_ receptor antagonist SR144528 were added 1 hour prior to incubation with anandamide. Values represent the mean ± SEM of 3 independent determinations, *P < .001. (c) The TRPV1 receptor antagonist SB366791 at 10 *μ*M was added 1 hour prior to incubation with anandamide. The data shown are average of two separate experiments ± range.

**Figure 3:**
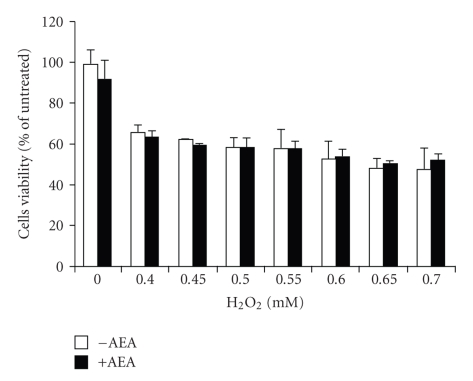
Effect of anandamide on H_2_O_2_ induced oxidative stress in PC12 cells. Cells were treated with 25 *μ*M anandamide for 24 hours, followed by treatment with the indicated concentrations of H_2_O_2_ for another 24 hours. Cell viability was assessed by the MTT assay. The data shown are average of two separate experiments ± range.

In order to determine whether the effect of anandamide is receptor-mediated, PC12 cells, which express the CB_1_ receptor ( [26], and our own data not shown) were incubated with selective CB_1_ or CB_2_ receptor antagonists, SR141716A or SR144528, respectively, prior to treatment with 25 *μ*M anandamide and 6-OHDA. The receptor antagonists did not inhibit the protective effects of anandamide, indicating that neither CB_1_ nor CB_2_ receptors are involved in anandamide protection against 6-OHDA toxicity (Figures 2(a) and 2(b)). Application of the antagonists at excess concentrations (25 *μ*M for SR141716A; 20 *μ*M for SR144528) also did not reverse the protection (Figures 2(a) and 2(b)). Since anandamide also has activity at the TRPV1 vanilloid receptor [8], and since this receptor is expressed in PC12 cells [26, 27], the effect of a TRPV1 selective antagonist, SB366791, on anandamide protection was examined. SB366791 had no effect on the protective abilities of anandamide against 6-OHDA-induced caspase activity (Figure 2(c)). In addition, none of the antagonists had any effect on 6-OHDA-induced DEVDase activity in the absence of anandamide. Cannabinoids, particularly those possessing a phenolic ring, are known to exert receptor-independent effects on cells and the neuroprotective effects have been suggested to be related to the potent antioxidant properties of cannabinoids [28]. Since 6-OHDA-induced cell death can be mediated by oxidative stress [17, 20, 29], the effect of anandamide on H_2_O_2_-induced cell death was examined. Anandamide did not prevent cell death due to H_2_O_2_ as determined by MTT survival assay (Figure 3). This suggests that anandamide-mediated protection against 6-OHDA is not a consequence of its antioxidant ability.

### 3.3. Involvement of PI3K and ERK1/2 Signalling Pathways in the Prosurvival Action of Anandamide

**Figure 4:**
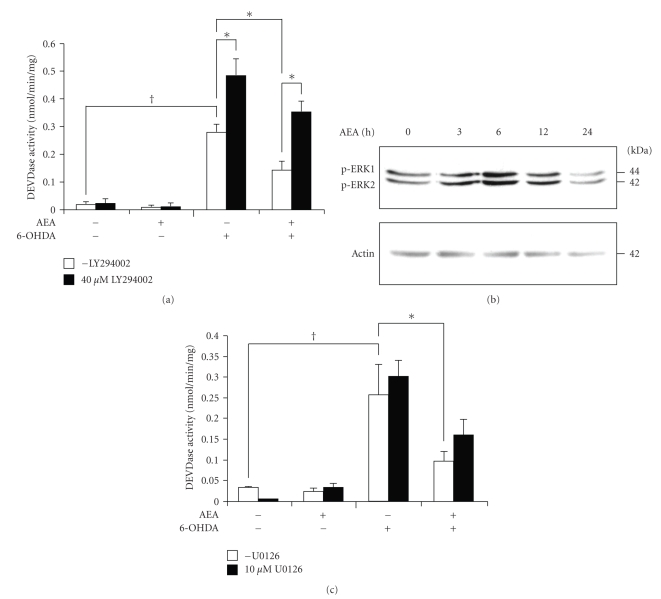
Role of PI3K and MAPK signalling in anandamide-mediated protection against 6-OHDA toxicity. (a) PC12 cells were pre-treated with 40 *μ*M LY294002 for 1 hours prior to addition of 25 *μ*M anandamide for 24 hours followed by treatment with 100 *μ*M 6-OHDA for a further 24 hours. Values represent the mean ± SEM of 4 independent experiments. *P < .005, *P* ^†^ < .001. (b) PC12 cells were treated with 25 *μ*M anandamide for 0–24 hours. The phosphorylation state of ERK1/2 was analysed by Western blotting. Actin protein levels were also analysed as a loading control. The results are representative of two separate experiments. (c) 10 *μ*M U0126, the MAPK pathway inhibitor was added 1 hour prior to incubation of PC12 cells with 25 *μ*M anandamide for 24 hours and with 100 *μ*M 6-OHDA for a further 24 hours. Values xrepresent mean ± SEM of three independent determinations. *P* ^†^ < .001, *P < .01.

Cannabinoid-dependent effects have been associated with several signal transduction pathways including activation of phosphatidylinositol 3-kinase (PI3K)/Akt and mitogen-activated protein kinase (MAPK) signalling pathways, both of which are linked to prosurvival signalling [30–32]. To gain insight into the molecular mechanism leading to cell survival, we studied the role of these signalling pathways in anandamide protection. Inhibition of PI3K with 40 *μ*M LY294002 was found to exacerbate 6-OHDA toxicity (Figure 4(a)). Pre-treatment with LY294002 reversed anandamide-mediated protection, although not to the level in the absence of anandamide, probably due to the effect of LY294002 in enhancing 6-OHDA-induced toxicity. 

To assess a possible role of MEK/ERK signalling in anandamide-mediated protection, we used Western blotting to examine the effect of anandamide on the phosphorylation status of ERK1/2. Treatment of PC12 cells with 25 *μ*M anandamide resulted in a transient increase in ERK1/2 phosphorylation at 6 hours that returned to basal levels by 24 hours (Figure 4(b)). Pharmacological inhibition of ERK activation by 10 *μ*M U0126 partially reversed the effect of anandamide on DEVDase activity, but without statistical significance (Figure 4(c)). This suggests that the increase in ERK1/2 phosphorylation does not make a major contribution to anandamide-mediated protection.

### 3.4. Anandamide Inhibits JNK Activation Induced by 6-OHDA

**Figure 5:**
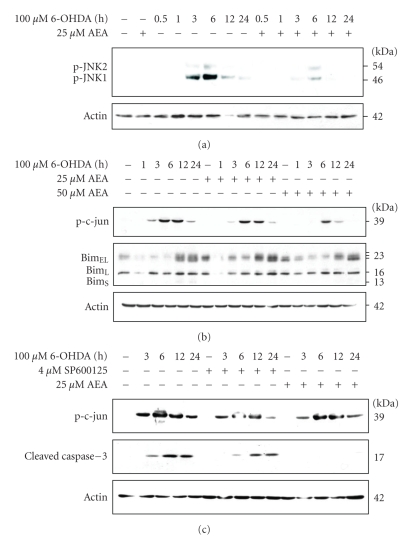
Anandamide partially protects against 6-OHDA-mediated apoptosis through inactivation of JNK signalling pathway. PC12 cells were treated with 25 *μ*M or 50 *μ*M anandamide for 24 hours and then exposed to 100 *μ*M 6-OHDA for indicated periods of time. (a) The phosphorylation state of JNK1/2 was analysed by Western blotting. Actin protein levels were also analysed as a loading control. (b) The phosphorylation state of c-Jun and Bim isoforms were analysed by Western blotting. Actin protein levels were also analysed as a loading control. Blots shown are representative of three independent experiments. (c) Where indicated, cells were exposed to 4 *μ*M SP600125 for 1 hour or 25 *μ*M AEA for 24 hour prior to treatment with 100 *μ*M 6-OHDA for further 24 hour. Phosphorylation of c-Jun and the processing of caspase-3 to the active 17 kDa fragment subunit were analysed by Western blotting. The levels of actin expression were also determined for loading control. The data shown are representative of two separate experiments.

The stress kinase JNK has been reported to mediate 6-OHDA-induced cell death [20, 33]. Therefore, we examined the effect of anandamide on JNK activation by examining the phosphorylation status of JNK1 and JNK2. Treatment of PC12 cells with 100 *μ*M 6-OHDA evoked a time-dependent increase in JNK1 phosphorylation with maximal activation after 6 hours (Figure 5(a)). There was a smaller increase in JNK2 phosphorylation that followed the same time course. Prior treatment with anandamide resulted in a marked inhibition of JNK1 phosphorylation (Figure 5(a)). These findings suggest that suppression of JNK activation may play an important role in anandamide-dependent protection against 6-OHDA and are in agreement with other studies showing that inhibition of JNK activity protects PC12 cells against 6-OHDA-induced apoptosis [20, 33–37]. 

Many JNK targets are implicated in cell death, including c-Jun and the BH3-only protein Bim [38]. Therefore, the effect of anandamide on these JNK targets was examined. 6-OHDA caused an increase in c-Jun phosphorylation which was reduced by anandamide pre-treatment (Figure 5(b)). Bim_EL_, a pro-apoptotic member of the Bcl-2 family, is also a target of JNK. Treatment of cells with 6-OHDA caused a marked increase in Bim_EL_ expression by 12 hours (Figure 5(b)). Three Bim_EL_ bands were observed with the higher molecular weight forms probably reflecting phosphorylated species, since this protein is known to be regulated by phosphorylation [39, 40]. In fact, we have previously shown that these upper bands represent phosphorylated Bim_EL_, since they disappear upon treatment of whole cell lysates with phosphatase [32]. Pre-treatment of the cells with anandamide did not appear to affect the time course or level of induction of Bim_EL_ (Figure 5(b)). However, there was a reduction in the higher molecular weight band for Bim_EL_ and an increase in the intensity of the lower molecular weight band at 24 hours of 6-OHDA with AEA treatment (Figure 5(b)). These data suggest that there is an anandamide-induced reduction in phosphorylation of Bim_EL_, possibly due to JNK inhibition.

In order to confirm a role for JNK activation in 6-OHDA toxicity, cells were pre-treated with the JNK inhibitor SP600125 (4 *μ*M). This inhibitor reduced phosphorylation of c-Jun by 6-OHDA and also caused a delay and a reduction in cleavage of caspase-3 to its active p17 fragment (Figure 5(c)). In the same experiment, 25 *μ*M anandamide had caused complete inhibition of caspase-3 cleavage while inhibiting c-Jun phosphorylation, but to a lesser extent than SP600125.

## 4. Discussion

Here we provide the first evidence for direct protective effects of anandamide against 6-OHDA toxicity. Anandamide blocks 6-OHDA-induced apoptosis in a concentration-dependent manner. This protection was upstream of caspase-3 activation since there was inhibition of DEVDase activity and a reduction in processing of pro-caspase-3. We were unable to demonstrate a role for CB_1_, CB_2_ or TRPV1 in anandamide protection. Nor did it involve ERK1/2 prosurvival signalling. However, a partial role for PI3K activation and suppression of JNK signalling were demonstrated. Cannabinoids have been shown to both induce and inhibit induction of cell death, (reviewed by [41]). Anandamide itself has been reported to be toxic [26] or nontoxic [42] to PC12 cells. In our study, anandamide, at the concentrations used, was not toxic even up to 48 hours of incubation (data not shown). Cannabinoid drugs have been shown by others to be protective in a variety of models of neurodegeneration [9, 41, 43], including neuronal cell death in models of Parkinson's disease [28]. For example, the non-selective synthetic cannabinoid receptor agonist HU210 was found to protect cerebellar granule neurons against 6-hydroxydopamine toxicity [28]. Furthermore, an in vivo study demonstrated that the plant-derived cannabinoids Δ^9^-THC and cannabidiol protect the rat nigrostriatal dopaminergic pathway from a medial forebrain bundle injection of the catecholamine neurotoxin 6-hydroxydopamine [28]. Although that in vivo study did not include direct assessment of neuroprotection, the ability of Δ^9^-THC or cannabidiol to ameliorate the effects of the 6-OHDA lesion on striatal dopamine concentration and tyrosine hydroxylase activity were shown. Our current data with anandamide support these earlier findings.

A possible role of metabolites of anandamide in the regulation of PC12 cell fate cannot be ruled out. Anandamide is enzymatically hydrolysed to ethanolamine and arachidonic acid [44]. In fact, this degradation has been shown to be necessary for its prosurvival effects in murine neuroblastoma cells where anandamide protective activity against serum deprivation was not mediated via  CB_1_/CB_2_ or TRPV1 receptors and required a breakdown of anandamide to ethanolamine [9]. 

It has been reported that induction of oxidative stress is involved in 6-OHDA-induced toxicity and antioxidants can protect against hydrogen peroxide and superoxide anion-mediated cell death [20, 33–37, 45–47]. To our knowledge, anandamide has not been reported to possess antioxidant activity. We did not observe anandamide-mediated protection of PC12 cells against H_2_O_2_-induced death, suggesting that anandamide does not inhibit oxidative stress due to 6-OHDA. However, H_2_O_2_ is not the only product of 6-OHDA auto-oxidation. In addition, superoxide anions and *p*-quinone, may contribute to reactive oxygen species (ROS) production [48–50] and may be inhibited by anandamide, thus blocking JNK activation and phosphorylation of its downstream targets. 

Cannabinoids have been shown to activate PI3K/Akt and MEK/ERK signalling pathways [51, 52]. While our results obtained with pharmacological inhibitors did not support a role for MEK/ERK signalling in anandamide protection against 6-hydroxydopamine, they did support an involvement of PI3K/Akt prosurvival signalling, since the PI3K selective inhibitor LY294002 reversed anandamide protection against 6-OHDA. In addition, the present data also support a possible role for inhibition of JNK activation in anandamide protection, since anandamide reduced 6-OHDA-induced JNK phosphorylation and JNK-dependent pro-apoptotic signalling, namely phosphorylation of c-Jun and possibly of Bim_EL_. 

JNKs are members of the mitogen-activated protein kinase (MAPK) pathway that is activated in response to many extracellular stimuli and different forms of environmental stress [53]. Our findings are in general agreement with recent reports showing that 6-OHDA induces JNK phosphorylation [36, 54] and that JNK activation mediates apoptosis induced by 6-OHDA in PC12 cells [45, 47, 54]. Furthermore, a number of studies showing protection by various agents against 6-OHDA neurotoxicity also show a concomitant suppression of JNK activation [20, 33–37]. We observed a large 6-OHDA-induced increase in phosphorylation of JNK1, and a minor increase in levels of phosphorylated JNK2 at 3–6 hours, which are accompanied by a concomitant increase in phospho-c-Jun and a delayed increase in the levels of Bim_EL_. Furthermore, the JNK inhibitor SP600125 caused a partial reduction in caspase-3 cleavage due to 6-OHDA. This was in contrast to complete inhibition by anandamide. These findings are in agreement with a previous report showing that SP600125 partially protects against 6-OHDA toxicity in PC12 cells [47]. Taken together, these data suggest a partial contribution of JNK signalling in the toxicity of 6-OHDA, and that anandamide may mediate some of its protective effect through suppression of this pathway.

JNK promotes apoptosis in a number of ways. Through phosphorylation and activation of c-Jun it stimulates the transcription of c-Jun target genes, including *Bim* [38]. In addition, JNK phosphorylates certain members of the Bcl-2 protein family, associated with the mitochondrial apoptotic pathway, including Bim_EL_, Bmf, and Bcl-2 [38, 53]. For example, Bim_EL_ levels and function are regulated by phosphorylation by ERK, JNK and possibly Akt [40, 55]. Phosphorylation of Bim_EL_ at Ser65 by active JNK potentiates its pro-apoptotic activity [56]. These data support an attractive hypothesis that anandamide protects against 6-OHDA at the level of, or upstream of, JNK activation. This inhibition of JNK by anandamide thus prevents the activation of downstream apoptotic pathways, including activation of c-Jun and possibly phosphorylation of Bim_EL_. However, since the level of cytoprotection by anandamide was far greater than that due to SP600125, despite similar JNK inhibition, it strongly suggests that anandamide utilises additional mechanisms to inhibit 6-OHDA toxicity. 

It should be considered that in contrast to the data supporting endocannabinoid-mediated JNK inhibition, there are data reported by others [57–59] indicating that cannabinoids induce JNK activation, an effect that precedes apoptotic events including caspase-3 activation and DNA fragmentation. Thus, the neuroprotective and neurotoxic effects of cannabinoids are likely to depend on a variety of factors, including toxic insults, doses and nature of cannabinoids (e.g., endocannabinoids versus phytocannabinoids), times of exposure and cell type [60, 61].

Here, for the first time we demonstrate that anandamide protects PC12 cells against 6-OHDA-induced apoptosis. These data and those of other studies may suggest potential therapeutic benefits of elevating anandamide and possibly other endocannabinoids in preventing the degeneration of nigrostriatal dopaminergic neurons in Parkinson's disease.
